# Intraoperative 3D imaging with cone-beam computed tomography leads to revision of pedicle screws in dorsal instrumentation: a retrospective analysis

**DOI:** 10.1186/s13018-021-02849-w

**Published:** 2021-12-04

**Authors:** Felix Zimmermann, Katharina Kohl, Maxim Privalov, Jochen Franke, Sven Y. Vetter

**Affiliations:** BG Klinik Ludwigshafen, Ludwig-Guttmann-Straße 13, 67071 Ludwigshafen am Rhein, Germany

**Keywords:** Spine, Dorsal instrumentation, Pedicle screw, Cone-beam CT, 3D imaging

## Abstract

**Background:**

Correct positioning of pedicle screws can be challenging. Intraoperative imaging may be helpful. The purpose of this study was to evaluate the use of intraoperative 3D imaging with a cone-beam CT. The hypotheses were that intraoperative 3D imaging (1) will lead to an intraoperative revision of pedicle screws and (2) may diminish the rate of perforated screws on postoperative imaging.

**Methods:**

Totally, 351 patients (age 60.9 ± 20.3 a (15–96); m/f 203/148) underwent dorsal instrumentation with intraoperative 3D imaging with 2215 pedicle screws at a trauma center level one. This study first evaluates intraoperative imaging. After this, 501 screws in 73 patients (age 62.5 ± 19.7 a; m/f 47/26) of this collective were included in the study group (SG) and their postoperative computed tomography was evaluated with regard to screw position. Then, 500 screws in 82 patients (age 64.8 ± 14.4 a; m/f 51/31) as control group (CG), who received the screws with conventional 2D fluoroscopy but without 3D imaging, were evaluated with regard to screw position.

**Results:**

During the placement of the 2215 pedicle screws, 158 (7.0%) intraoperative revisions occurred as a result of 3D imaging. Postoperative computed tomography of the SG showed 445 (88.8%) screws without relevant perforation (type A + B), of which 410 (81.8%) could be classified as type A and 35 (7.0%) could be classified as type B. Fifty-six (11.2%) screws in SG showed relevant perforation (type C–E). In contrast, 384 (76.8%) screws in the CG were without relevant perforation (type A + B), of which 282 (56.4%) could be classified as type A and 102 (20.4%) as type B. One hundred and sixteen (23.2%) screws in the CG showed relevant perforation (type C–E).

**Conclusion:**

This study shows that correct placement of pedicle screws in spine surgery with conventional 2D fluoroscopy is challenging. Misplacement of screws cannot always be prevented. Intraoperative 3D imaging with a CBCT can be helpful to detect and revise misplaced pedicle screws intraoperatively. The use of intraoperative 3D imaging will probably minimize the number of revision procedures due to perforating pedicle screws.

## Background

The current gold standard for the surgical therapy of spinal fractures, in degenerative and in the majority of congenital diseases, is the dorsal instrumentation of the spine using pedicle screws [1, 2]. One difficulty in inserting screws in the spine is their correct placement in the pedicle without perforating medially or laterally. Misplaced pedicle screws after dorsal instrumentation are observed in between 1.5 and 40% of cases in the current literature [3–5]. However, this is of particular importance due to the adjacent structures. Ideally, the screw should be centrally located in the pedicle without tangentially affecting or perforating the pedicle cortex. While lateral malpositioning of the pedicle screw may affect the stability, medial perforation of the pedicle screw may be associated with severe neurologic impairment. Despite this anatomic proximity, injuries of these important nervous structures are rarely observed. Esses et al. observed temporary neurologic impairment in 2.4% and persistent neurologic damage in 2.3% of their 617 surgically treated patients [5]. Moreover, the screw tip should lie in the vertebral body, but without penetrating the ventral cortex. This is also very important if adjacent organs (e.g., aorta, vena cava, lung, trachea, esophagus, etc.) and blood vessels are present, which can be injured intraoperatively. Fortunately, this rarely happens [3, 6–8].

Consequently, it is necessary to check and assess the pedicle screw position intraoperatively, for which Gertzbein–Robbins et al. developed their classification (A–E) of pedicle screw position [9]. Deviations > 2 mm outside the pedicle are classified as type C according to Gertzbein–Robbins [9] and are considered to need correction [10], even if no neurologic damage is to be expected with medial deviations up to 4 mm [11]. At the same time, however, it must be taken into account that the thoracic pedicles are commonly < 4 mm wide, so that even thin pedicle screws can perforate the pedicle in this area [12]. Even though the Gertzbein–Robbins classification facilitates the assessment of the screw position, the practical implementation in the operating room with the aid of conventional 2D fluoroscopy remains highly challenging or, in some cases, gives great residual uncertainty. Therefore, computed tomography is recommended to assess screw positioning and reduction [1, 11, 13, 14]. Intraoperative availability of computed tomography is low. Screw malpositioning or inadequate reduction detected on postoperative computed tomography may necessitate revision surgery. Accordingly, intraoperative 3D imaging with cone-beam CT (CBCT) has been developed to close this gap and to facilitate intraoperative assessment. The advantage of CBCT imaging has been demonstrated in other anatomic regions [15]. The purpose of this retrospective study was to evaluate the intraoperative revision rate, revision reason, and postoperative result of pedicle screw implantations after 3D imaging with CBCT. The hypotheses were that the use of intraoperative 3D imaging to analyze the pedicle screw placement in dorsal instrumentation (1) will lead to intraoperative revision of pedicle screws and (2) may reduce the rate of misplaced screws on postoperative imaging.

## Methods

This study obtained approval from the local ethics committee (Reference no. 2020-15452-retrospektiv). A total of 351 patients (age 60.9 ± 20.3 (15–96) y; male/female 203/148) underwent dorsal instrumentation with intraoperative 3D imaging at a trauma center level one between January 2013 and August 2020. The patients were placed in the prone position during surgery. The pedicle screws were inserted percutaneously through small skin incisions in free-hand technique cannulated over pre-inserted Kirschner wires (K-wires). Intraoperatively, the K-wires or screw position and reduction were assessed under 2D fluoroscopy as usual. Only if the results were satisfactory, 3D imaging with a CBCT was performed. Two different CBCT models were used for this: Cios Spin (Siemens, Erlangen, Germany) and ARCADIS Orbic 3D (Siemens, Erlangen, Germany). While Cios Spin is a 3D C-arm with a flat panel, the ARCADIS Orbic is a 3D fluoroscopy with an image intensifier‐based 3D imaging C‐arm.

Technically, these devices rotate around the spine and automatically capture a series of 2D images. Image generation on the ARCADIS Orbic is based on analog image intensifier technology, with limitations in the area of acquired image data, contrast differences, and susceptibility to artifacts. Since pedicle screws are relatively large compared to the anatomic structures being examined, artifacts can significantly complicate image assessment. Accordingly, to improve image quality and reduce susceptibility to artifacts, new flat panel detectors have been developed and are used in the newer flat panel C-arm generations. [16].

Coronal, sagittal, and axial projections allowed the reduction and placement of screws to be evaluated. Image assessment can be performed either slice by slice or on a reconstructed 3D model, as usual when assessing CT imaging.

The CBCT scans obtained were evaluated immediately after imaging. Pedicle screw location was assessed using the Gertzbein–Robbins classification [9] modified from Schatlo et al. [17]. Pedicle screws with a deviation of more than 2 mm (Gertzbein–Robbins type C–E) according to Schatlo et al. [17] outside the pedicle were classified as perforated and in need of intraoperative revision. The findings were documented intraoperatively in an Excel spreadsheet (Microsoft, Redmond, USA) immediately after evaluation. If malpositioned pedicle screws (Gertzbein–Robbins type C–E [17]) were detected in the CBCT scans, they were corrected accordingly. And in the case of immediate intraoperative revision, the same sequence of intraoperative imaging was followed as before: 2D fluoroscopy and then intraoperative 3D imaging. The collected findings were then reassessed and documented. This procedure was continued until a satisfactory result was determined in the intraoperative 3D imaging.

A pedicle perforating screw placement with dislocation > 2 mm (Gertzbein–Robbins type C–E) was a reason for surgical revision.

Postoperative computed tomography (Aquilion Prime SP and Aquilion Lightning, Canon, Tokyo, Japan) was performed to verify the surgical outcome.

As a control group (CG), 82 patients (age 64.8 ± 14.4 a; m/f 51/31) with a total of 500 screws, who received dorsal instrumentation with conventional fluoroscopy without 3D imaging in the same time period, were included.

**Fig. 1:**
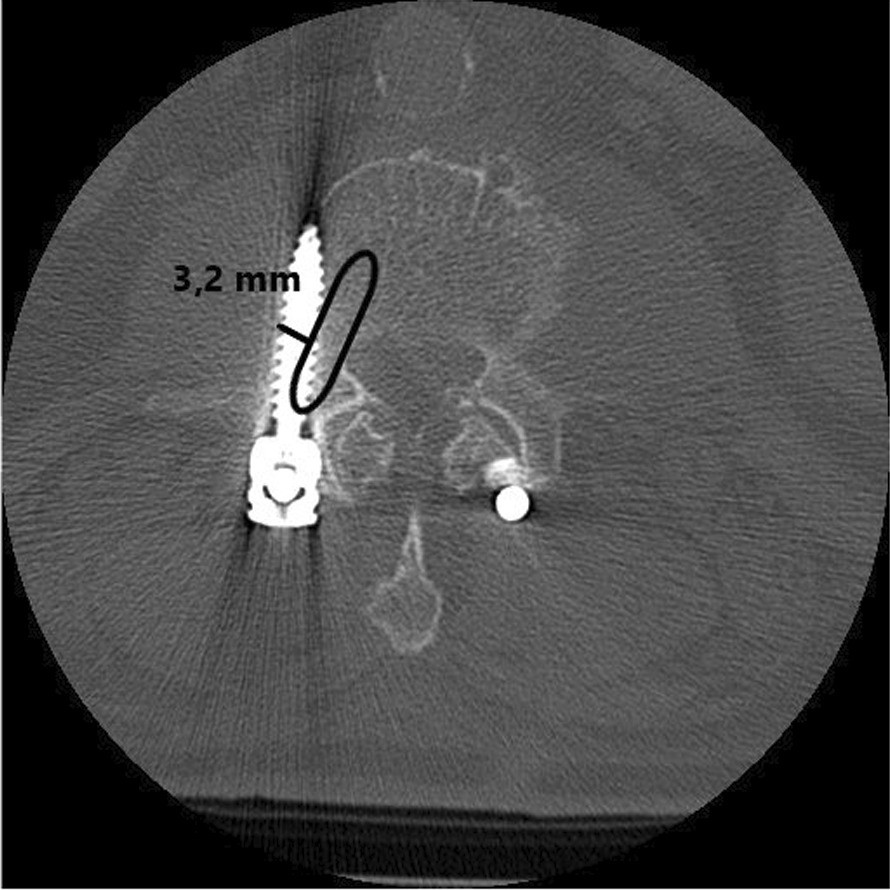
Assessment of the pedicle screw position using the Gertzbein–Robbins–Robbins classification [9]. Marking of the bony pedicle borders by an ellipse. Then determine the maximum screw distance from the (in this case lateral) pedicle border

In this study, we retrospectively evaluated the intraoperatively created database of the intraoperative 3D imaging with CBCT. After that, we evaluated the postoperative computed tomography results of SG and CG. In both groups, we analyzed the pedicle screw position on postoperative computed tomography using Gertzbein–Robbins–Robbins classification [9] modified from Schatlo et al. [17] (Fig. 1).

The normality of the continuous data was assessed, and the data are presented as the means ± standard deviations (ranges). Wilcoxon tests were used to assess the differences between the study group and the control group. All analyses were performed using SAS. The significance level was set at 0.05.

## Results

During the placement of the 2215 pedicle screws in a total of 351 patients between January 2013 and August 2020, 437 CBCT scans were performed. Eighty-six patients received multiple intraoperative CBCT scans, with a maximum of two CBCT scans in one surgery. In all patients, the intraoperative 3D imaging was performed for diagnostic reasons and not due to complications. In summary, a total of 158 (7.0%) intraoperative revisions were made intraoperatively based on the intraoperative 3D imaging (Table 1).

**Table 1:** Revision numbers of pedicle screws in relation to the number of scans of intraoperative cone-beam CT imaging in 2215 pedicle screws

	After scan 1	After scan 2	Total
Revised pedicle screws	134 (6.1%)	23 (1.0%)	158 (7.0%)

Evaluation of postoperative computed tomography of the SG showed non-relevant perforating of the screws (Gertzbein–Robbins type A + B) in 445 (88.8%) cases, of which 410 (81.8%) cases could be classified as type A and 35 (7.0%) cases could be classified as type B. Fifty-six (11.2%) of 501 pedicle screws showed relevant perforation of the pedicle (Gertzbein–Robbins type C–E).

In contrast, 384 (76.8%) screws in the CG were without relevant perforation (Gertzbein–Robbins type A + B), of which 282 (56.4%) could be classified as type A and 102 (20.4%) screws as type B. One hundred and sixteen (23.2%) screws in CG showed relevant perforation (Gertzbein–Robbins type C–E).

The majority of pedicle screws in both SG and CG were inserted in the thoracic segments of the spine (Table 2).

**Table 2:** Absolute and relative distribution of pedicle screws of the study (SG) and control group (CG) on the spinal segments

	Cervical segment	Thoracic segment	Lumbar segment
SG (*n* = 501)	159 (31.7%)	250 (49.9%)	92 (18.4%)
Type C–E of SG (*n* = 56)	7 (12.5%)	36 (64.3%)	13 (23.2%)
CG (*n* = 498)	31 (6.2%)	247 (49.6%)	220 (44.2%)
Type C–E of CG (*n* = 116)	3 (2.6%)	69 (59.5%)	44 (37.9%)

The difference between the numbers of screws without relevant perforation (Gertzbein–Robbins type A + B) in the SG and CG was statistically significant (*p* < 0.0001). However, there was no statistically significant difference between the numbers of screws with relevant perforation in the SG and CG (*p* < 0.8791).

Of the 56 screws in SG with relevant pedicle perforation (Gertzbein–Robbins type C–E) on postoperative computed tomography, 26 screws showed medial deviation (2.9 ± 0.9 mm) and 29 screws showed lateral deviation (3.0 ± 1.1 mm) from the pedicle. One screw breached out caudally (2 mm). No screw showed cranial deviation.

In the postoperative imaging of the 116 pedicle perforating screws of the CG in the postoperative imaging, 45 screws showed medial deviation (2.6 ± 0.6 mm) and 70 screws showed lateral deviation (3.2 ± 1.2 mm) from the pedicle. Again, one screw breached out caudally (2 mm) and no screw showed a cranial deviation.

## Discussion

The purpose of this retrospective study was to evaluate the intraoperative revision rate because of 3D imaging and postoperative result of pedicle screw implantations in dorsal instrumentation with and without 3D imaging. The hypotheses were that the use of intraoperative 3D imaging as part of dorsal instrumentation in spine surgery (1) will lead to intraoperative revision of pedicle screws and (2) may reduce the rate of misplaced screws on postoperative imaging.

In our study, a total of 158 (7.0%) of 2215 implanted pedicle screws were not correctly positioned using conventional 2D fluoroscopy-assisted dorsal instrumentation and were revised as a result of intraoperative 3D imaging with cone-beam CT during the primary procedure. On postoperative computed tomography, 56 (11.2%) of 500 implanted pedicle screws in SG with intraoperative 3D imaging were still with relevant pedicle perforation (Gertzbein–Robbins type C–E). Conversely, in CG without intraoperative 3D imaging 116 (23.2%) screws remained with relevant pedicle perforation. Accordingly, intraoperative 3D imaging may reduce the rate of misplaced pedicle screws on postoperative CT imaging.

Assessment of pedicle screw placement in dorsal instrumentation on conventional 2D fluoroscopy imaging is a major challenge even for the experienced surgeon [13]. Berlemann et al. were able to show that 59% of all incorrectly positioned pedicle screws are missed in 2D fluoroscopy imaging compared with computed tomography imaging [14]. Accordingly, computed tomography can be seen as the gold standard in the assessment of pedicle screw location [1, 11, 13, 14]. However, if a finding requiring revision only becomes visible on postoperative computed tomography, then a revision operation is necessary. This can be circumvented with intraoperative 3D imaging. The availability of intraoperative computed tomography is low, so that 2D methods, from which 3D images can be calculated, are increasingly used intraoperatively to close this gap. This includes cone-beam CT (CBCT). The positive effect of the intraoperative application of CBCT has already been demonstrated in other anatomic regions [15].

Three different methods of implanting pedicle screws are described in the current literature: free-hand technique, with the aid of fluoroscopy, or in a navigated technique.

Malposition rates (1.7–31%) in postoperative computed tomography with free-hand technique can be significant [18, 19], whereas the use of conventional 2D fluoroscopy to check screw position intraoperatively may not improve the rate of screws fully contained in the pedicle (28–85%) [18]. To prevent screw misplacement, navigation systems have been developed using 3D fluoroscopic or CT imaging to achieve better results [20, 21]. Revision rates because of misplaced screws have also been reduced by 3D navigation. This is illustrated by studies that discovered three times higher revision rates for non-navigated techniques compared to 3D navigation [20]. Both CT-navigated and fluoroscopy-navigated techniques show better postoperative results. In the review by Gelalis et al., rates of 89–100% and 81–92%, respectively, of screws fully contained in the pedicle were reported [18]. Moreover, Takahata et al. implanted a total of 166 CBCT-navigated pedicle screws in a collective of 48 consecutive patients with a rate of 2.4% misplaced pedicle screws [21]. These data indicate that intraoperative 3D navigation provides greater accuracy in pedicle screw insertion than the free-hand technique and conventional 2D fluoroscopy.

Advantages of the non-navigated technique with intraoperative 3D imaging generated with CBCT are that it is more readily available and less expensive compared to the navigated technique. In addition, compared to the non-navigated technique, preparation of the navigated technique in surgery extends the operating time by a few minutes [22]. 3D imaging with CBCT also extends the operating time—even if only slightly—compared to conventional fluoroscopy, which has already been shown in the current literature [23, 24]. In the context of this study, the time of the CBCT examination was not explicitly investigated, but was determined in an in-house investigation earlier which showed that the procedure is extended by approximately 5 min due to 3D imaging. Furthermore, the non-navigated technique with intraoperative 3D imaging does not have such a flat learning curve as the navigated technique, so it can be integrated into the clinical routine more quickly.

Little data are available in the literature concerning the non-navigated technique supported with 3D imaging with CBCT that we use. Cordemans et al. performed a study to verify pedicle screw position with intraoperative 3D imaging using CBCT. They implanted a total of 695 screws in 118 patients. The rate of pedicle breaching screws on intraoperative CBCT was 11.7% in their collective [25].

In addition, further studies demonstrated that intraoperative 3D imaging with a CBCT is not inferior to established computed tomography. The authors concluded that intraoperative CBCT could replace postoperative computed tomography after dorsal instrumentation in the future [26, 27].

A disadvantage of 3D imaging with CBCT compared to conventional fluoroscopy is the higher radiation exposure caused by 3D imaging [28], which is certainly not negligible. Nevertheless, in our opinion, the advantage of the increased accuracy of screw positioning is superior to the disadvantage of the increased radiation exposure compared to conventional fluoroscopy.

Based on the 3D imaging generated with the CBCT, a sufficient assessment of the pedicle screw position can be made and a not-to-be-underestimated number of perforating screws can be detected intraoperatively, as is evident from our results and the current literature. Accordingly, in our view, intraoperative 3D imaging with a CBCT in the context of dorsal instrumentation is a good alternative to the navigated technique or postoperative computed tomography with regard to the radiologic results of pedicle screw position.

However, the results of this study must be interpreted in light of several limitations: (1) This is a retrospective evaluation of intraoperatively generated 3D imaging. (2) Only the radiologic results of intraoperative 3D imaging were assessed but not the clinical outcomes, and so it is not clear to what extent a nonoptimal pedicle screw position in the postoperative computed tomography affects the outcome. However, since none of the misplaced screws were revised following postoperative CT imaging, it can be assumed that they were not clinically relevant. (3) In the study, two different CBCTs were used for intraoperative 3D imaging: Cios Spin (Siemens, Erlangen, Germany) and ARCADIS Orbic 3D (Siemens, Erlangen, Germany). The quality of imaging acquired from the Cios Spin was superior to that of the ARCADIS Orbic 3D. Even though the radiologic evaluation of the imaging did not show any influence of the type of CBCT on the result, a certain influence cannot be excluded with absolute certainty. In our view, the ethical duty to provide optimal patient care made it impossible to omit intraoperative 3D imaging for pure study reasons.

## Conclusion

This study shows that correct placement of pedicle screws in spine surgery with conventional 2D fluoroscopy is challenging. Misplacement of screws cannot always be prevented. Intraoperative 3D imaging with a CBCT can be helpful to detect and revise misplaced pedicle screws intraoperatively. The use of intraoperative 3D imaging will probably minimize the number of revision procedures due to perforating pedicle screws.

## Data Availability

The datasets used and analyzed during the current study are available from the corresponding author on reasonable request.
